# Verapamil and hematoporphyrin derivative for tumour destruction by photodynamic therapy.

**DOI:** 10.1038/bjc.1991.244

**Published:** 1991-07

**Authors:** L. Gossner, H. Wittke, A. Warzecha, R. Sroka, H. Ernst, M. Meier, C. Ell

**Affiliations:** Department of Medicine, University of Erlangen-Nuremberg, Germany.


Br. J. Cancer (1991), 64, 84–86 © Macmillan Press Ltd., 1991

SHORT COMMUNICATION

### Verapamil and hematoporphyrin derivative for tumour destruction by photodynamic therapy

L. Gossner[1], H. Wittke[1], A. Warzecha[1], R. Sroka[1,2], H. Ernst[1], M. Meier[2] & C. Ell[1]

[1]*Department of Medicine, University of Erlangen – Nuremberg, D-8520 Erlangen;* [2]*GSF – Zentrales Laserlaboratorium, D-8042 Neuherberg/Munich, Germany.*

In recent years, photodynamic therapy (PDT) has shown much promise for the local and selective destruction of malignant tumours. Although tumour destruction is believed to be mediated through the production of highly reactive intermediate singlet oxygen by photoactivated hematoporphyrins (Weishaupt *et al.*, 1986), considerable evidence has accumulated to suggest that the primary site of photodynamic damage is the small vessels and capillaries of the tumours (Nelson *et al.*, 1988; Berenbaum *et al.*, 1986). Some studies have shown vascular effects occurring with PDT such as the fall of tumour · blood flow (Selman *et al.*, 1984; Wieman *et al.*, 1988) and the shutdown of tumour vessels (Henderson *et al.*, 1985). In one report a complete cessation of tumour blood flow was described in rat tumours after PDT (Star *et al.*, 1986). In solid tumours the drug uptake is limited by the tissue perfusion rate, the membrane permeability and the transport across the vessel wall (Gerlowski *et al.*, 1986). Therefore, it seemed plausible that vasoactive drugs might influence tumour destruction by PDT. In particular the calcium channel blockers have generated much interest in cancer research since it has been demonstrated that verapamil, the prototype calcium channel blocker, increases the cytostatic effects of adriamycin and vincristine (Tsuruo *et al.*, 1983) and has a reversible antiproliferative effect itself (Schmidt *et al.*, 1988). Although the precise mechanism of action is not known, some studies indicate that verapamil inhibits the P-glycoprotein pump which drug-resistant tumour cells use to pump out anticancer agents (Ince *et al.*, 1986; Garman *et al.*, 1983).

Recently an enhanced photodynamic destruction of tumours was described when verapamil was concurrently administered with the photosensitiser, or similarly when verapamil was injected after PDT, a delay of the regrowth of tumours was implicated (Cowled & Forbes, 1989). In contrast to these authors, who administered high doses of hematoporphyrin derivative (HPD, 30–50 mg kg$^{-1}$ body weight), we injected doses of hematoporphyrin derivative enriched with dihematoporphyrin-ether (DHE, 1.5 or 9 mg kg$^{-1}$ body weight) according to previous experiments (Sroka *et al.*, 1989*a*). In our experiments we used two different tumour models to examine the effects of verapamil on the photodynamic destruction of tumours.

Our first tumour model, the isogeneic fibrosarcoma SSK-2 was implanted into the flank of female inbred C3H-mice. This fibrosarcoma grows with a doubling time of approximately 1.5 days. The tumour size was measured with calibration masks, gauged to the weight of tumours (Kummermehr & Trott, 1982). The photodynamic efficiency was quantified by means of the tumour regrowth delay time, i.e. the time a tumour needs to regain a defined weight (Begg, 1980). When the tumours reached a weight of 60 mg, the photosensitiser and verapamil, both diluted with saline solution, were injected intraperitoneally at a dose of 9 mg kg$^{-1}$ body weight according to previous experiments (Stocker, 1986). The values of the individual regrowth delays were plotted and, assuming their Gaussian statistical distribution, approximated in a least square fit procedure by an exponential curve. Mean values and standard deviations of the regrowth delay have been calculated for a clearer presentation. In addition, the extent of tumour necrosis after PDT was examined histologically in a second group of tumour bearing mice in order to compare the regrowth delay with the tumour necrosis.

Our second *in vivo* model was a human adenocarcinoma of the colon, heterotransplanted with the standard technique (Sroka *et al.*, 1989*b*) into nude mice. When the tumours reached a 1 cm diameter, the drugs were administered and the tumours were irradiated.

Five days after PDT the mice were sacrificed and the tumours were resected. The percentage of the tumour necrosis was evaluated histologically by three independent examiners. Mean values and standard deviations of the tumour necroses were calculated (SAS users guide: Basics and statistics, 1985).

In both murine tumour models and animals were anaesthetised (Inhalation narcosis with Enfluran: Ethrane, Abbot GmbH, FRG) during the time of irradiation. The mice were divided into six groups:

| *Group* | *Treatment* |
|---|---|
| A | No drugs, no light, typical growth/spontaneous necrosis. |
| B | Only photosensitiser administered (DHE). |
| C | Only verapamil administered. |
| D | Only light without drugs. |
| E | Photosensitiser and light administered (PDT). |
| F | Photosensitiser + verapamil and light administered. |

In each group, ten animals were treated per experiment. All experiments were repeated twice so that a total of 30 mice were treated in each group.

Photosan 3 (Seehof Laboratory, FRG), a hematoporphyrin derivative enriched with dihematoporphyrinether (DHE), was administered intraperitoneally to the animals at a concentration of 1.5 mg kg$^{-1}$ (human adenocarcinoma and fibrosarcoma SSK-2) and 9 mg kg$^{-1}$ (fibrosarcoma SSK-2) body weight.

Verapamil (Isoptin, Knoll AG, FRG), formulated for clinical use, was injected concurrently with the photosensitiser at a dose of 2 mg kg$^{-1}$ body weight. Twenty-four hours after application of of the drugs, the tumours were irradiated with laser light.

Tumours were treated with laser light tuned to the wavelength of 630 nm. The radiation was delivered from an Argon-ion laser-pumped dye laser (model 171 and 375 B, Spectraphysics Inc., USA; Dye: Kiton red). A tube, covering the tumour, was fed by a flexible quartz fibre (core diameter: 600 μm) and guaranteed nearly homogenous irradiation due

Correspondence: L. Gossner, Department of Medicine, University of Erlangen – Nuremberg, Krankenhausstr. 12, D-8520 Erlangen, Germany.
Received 14 September 1989; and in revised form 4 February 1991.

to multiple inner reflection (Sroka *et al.*, 1989*b*). The total energy density was 150 J $cm^{-2}$ at a power density of 400 mW $cm^{-2}$. The tube was cooled by a flow of $N_2$ gas in order to avoid hyperthermic effects at this high power density. With gas cooling, a maximum temperature of 38°C was recorded. The temperature was measured subcutaneously between skin and tumour.

In the fibrosarcoma SSK-2 tumour model the photosensitiser and verapamil were concurrently administered and tumours were irradiated 24 h later. Both, regrowth delay time and extent of tissue necrosis of the treated tumours were examined. The results are shown in Table I and II. These data show that verapamil did not enhance photodynamic destruction of tumours. This drug did not markedly affect the regrowth delay time measured in the fibrosarcoma. The comparison of tumours treated with PDT plus verapamil (group F) and PDT alone (group E) showed no effective inhibition of tumour regrowth. Verapamil alone (group C) and DHE without irradiation (group B) did not affect the regrowth delay time.

With respect to the percentage of necrosis measured (Table II) the tumours demonstrated a similar behaviour in each group. A concentration of 9 mg $kg^{-1}$ body weight Photosan 3 showed a subtotal destruction of the tumour by PDT alone. Therefore, the photosensitiser dosage was reduced to 1.5 mg $kg^{-1}$ body weight. At this concentration tumour control was less effective: There was a significant reduction of tumour necrosis to 18%. Verapamil plus PDT did not increase the amount of tumour tissue necrosis in both cases. Verapamil alone (group C) did not affect the tumours macro- or microscopically.

Our second *in vivo* model involves tumours of human adenocarcinoma of the colon which were transplanted into nude mice. In this model we examined the effects of verapamil and PDT on tumour destruction alone (Table III). Under the conditions tested, verapamil did not enhance the photodynamic destruction of the human colon carcinoma. Verapamil plus PDT had no effect on the degree of tumour tissue necrosis when compared to PDT alone. The extent of tumour necrosis was not influenced by verapamil alone (group C), DHE alone (group B) or light without drugs (group D) compared to controls (group A).

The process in which tumour damage is caused by photodynamic therapy is complex dependent on many different factors. Experimental studies have shown that the most important parameters are the applied energy density, the concentration of the administered photosensitiser in the tissue and the time interval between irradiation and administration of the photosensitiser (Barr *et al.*, 1989; Potter *et al.*, 1987). The photosensitiser uptake and thus the concentration in tissue are thought to be affected by the tissue perfusion rate. Therefore, the influence of vasoactive drugs such as verapamil on PDT was examined in recent studies. Cowled and Forbes described an increased photodynamic destruction of tumours with verapamil by using a transplantable tumour model in mice. In contrast to doses of 30–50 mg $kg^{-1}$ HPD as used by Cowled and Forbes, low photosensitiser doses were applied according to previous experiments which proved to be sufficient to cause a subtotal tumour destruction (Gossner *et al.*, 1991). Higher drug doses did not enhance the amount of tumour destruction, only the danger of adverse phototoxic side-effects could possibly increase. Even if these dosage schedules cannot easily be transferred to clinical application, it seems to be clear that the lowest possible photosensitiser concentration should be applied to avoid phototoxic side-effects of the skin (Wooton *et al.*, 1988).

In view of the results found in our two different tumour models, we conclude that verapamil does not increase photodynamic damage concurrently administered with low doses of DHE in our *in vivo* models. It could be demonstrated that for a low photosensitiser concentration neither a regrowth delay nor an increased extent of tumour tissue necrosis is achieved. However, in other studies intracellular concentrations of cytotoxic agents such as adriamycin and vincristine were increased, suggesting that verapamil improved uptake and inhibited transport of drugs through the cell membrane (Tsuruo *et al.*, 1983). Thus the supposed pharmacological mechanism is the existence of a drug elimination pathway in the plasma membrane of cancer cells. A possible explanation could be the concept that verapamil blocks the P-glycoprotein pump which tumour cells use to transport anticancer drugs out of the cell (Ince *et al.*, 1986). But a certain intracellular concentration of the applied drug has to be reached to activate the P-glycoprotein mechanism.

It is known that the photosensitiser concentration ratio between tumour and normal tissue is only 2.5:1 (Barr *et al.*, 1989). In accordance with the P-glycoprotein mechanism hypothesis, this photosensitiser concentration could be too low to trigger this drug elimination pathway and might be the reason why we did not find an enhanced destruction of

**Table I** Fibrosarcoma SSK-2: Effect of verapamil concurrently administered with DHE on tumour growth time and regrowth delay time

| | Animals | Growth time (d) | | Regrowth delay (d) | |
|---|---|---|---|---|---|
| | (n) | Mean | s.d. | Mean | s.d. |
| A Control | 30 | 6.0 | 1.5 | 0 | 0 |
| B DHE alone | 30 | 6.6 | 0.9 | 0 | 0 |
| C Verapamil alone | 30 | 6.5 | 1.5 | 0 | 0 |
| D Light alone (without drugs) | 30 | 5.6 | 1.1 | 0 | 0 |
| E DHE + light (PDT) | 30 | 16.5 | 3.6 | 10.5 | 3.6 |
| F PDT + verapamil | 30 | 17.4 | 3.8 | 11.4 | 3.8 |

Differences between A through D and E, F are significant ($P < 0.05$).

**Table II** Fibrosarcoma SSK-2: Influence of verapamil concurrently administered with DHE on the extent of tumour necrosis

| | Animals | Tumour necrosis (%) | |
|---|---|---|---|
| | (n) | mean | s.d. |
| A Control | 30 | 4.2 | 4.0 |
| B DHE alone | 30 | 5.0 | 3.4 |
| C Verapamil alone | 30 | 2.8 | 1.0 |
| D Light alone (without drugs) | 30 | 4.8 | 3.2 |
| E DHE + light (PDT) | 30 | 95.3 | 1.1 |
| | 30[a] | 18.0 | 1.6 |
| F PDT + verapamil | 30 | 92.1 | 6.3 |
| | 30[a] | 19.8 | 5.4 |

Differences between A through D and E, F are significant ($P < 0.05$).
[a]With a photosensitiser concentration of 1.5 mg $kg^{-1}$ body weight.

**Table III** Human adenocarcinoma of the colon: Effect of verapamil concurrently administered with DHE on photodynamic tumour destruction

| | Animals (n) | Tumour necrosis (%) mean | s.d. |
|---|---|---|---|
| A Control | 30 | 36.1 | 10.5 |
| B DHE alone | 30 | 38.7 | 8.5 |
| C Verapamil alone | 30 | 47.7 | 4.4 |
| D Light alone (without drugs) | 30 | 42.4 | 10.0 |
| E DHE + light (PDT) | 30 | 67.5 | 6.6 |
| F PDT + verapamil | 30 | 70.1 | 10.0 |

Differences between A through D and E, F are significant ($P < 0.05$).

malignant tissue by verapamil.

Cowled and Forbes used a different tumour model with different drug concentrations. Therefore, the question remains to be solved whether the lower photosensitiser concentration or the type of tumour tested is the reason why we did not find an enhanced tumour destruction in combination with verapamil. From our results it seems to be clear that there is no generality in the phenomenon described by Cowled and Forbes.

In spite of our negative experiments, the possible enhancement of photodynamic destruction of tumours by vasoactive drugs deserve further investigations. In a recent study, norverapamil, a mayor metabolite of verapamil with no systemic side effects, has proved to be as effective as verapamil (Merry *et al.*, 1989) offering new possibilities in testing vasoactive drugs and photodynamic therapy.

For low dose administration of DHE, our current experimental strategies comprise different potential modifiers such as the application of glucose (Thomas & Girotti, 1989) or improved targeting with liposomes (Jori *et al.*, 1986) or monoclonal antibodies (Mew *et al.*, 1983).

The authors thank G. Nowak for her helpful assistance and the Wilhelm-Sander Stiftung for financial support (grant No. 85.001.2).

### References

BARR, H., BROWN, S.G., KRASNER, N. & BOULOS, P.B. (1989). Photodynamic therapy for colorectal disease. *Int. J. Colorect. Dis.*, **4**, 15.

BEGG, A.C. (1980). Analysis of growth delay data: potential pitfalls. *Br. J. Cancer*, **41**, 93.

BERENBAUM, M.C., HALL, G.W. & HOYES, A.D. (1986). Cerebral photosensitisation by haematoporphyrin derivative: evidence for an endothelial site of action. *Br. J. Cancer*, **53**, 81.

COWLED, P.A. & FORBES, I.J. (1989). Modification by vasoactive drugs of tumour destruction by photodynamic therapy with hematoporphyrin derivative. *Br. J. Cancer*, **59**, 90.

GARMAN, D., ALBERS, L. & CENTER, M.S. (1983). Identification and characterisation of a plasma membrane phosphoprotein which is present in chinese hamster lung cells resistant to adriamycin. *Biochem. Pharmacol.*, **32**, 3633.

GERLOWSKI, L.E. & JAIN, R.K. (1986). Microvascular permeability of normal and neoplastic tissues. *Microvasc. Res.*, **31**, 288.

GOSSNER, L., WITTKE, H., ERNST, H., LEBEK, R., SROKA, R. & ELL, C. (1991). Photodynamische Therapie humaner Kolonkarzinome: substanzdosis- und Energiedichteabhängigkeit im thymusaplastischen Nacktmausmodell. In *Aktuelle Therapie gastrointestinaler Tumoren*, Schmoll, H.J. & Pichlmayr, R. (eds) p. 261. Springer: Berlin (in press).

HENDERSON, B.W., WALDOW, S.M., MANG, T.S., POTTER, R.W., MALONE, P.B. & DOUGHERTY, T.J. (1985). Tumor destruction and kinetics of tumor cell death in two experimental mouse tumors following photodynamic therapy. *Cancer Res.*, **45**, 1924.

INCE, P., APPLETON, D.R., FINNEY, K.J., SUNTER, J.P. & WATSON, A.J. (1986). Verapamil increases the sensitivity of primary human colorectal carcinoma tissue to vincristine. *Br. J. Cancer*, **53**, 137.

JORI, G., REDDI, E., COZZONI, J. & TOMIO, L. (1986). Controlled targeting of different subcellular sites by porphyrins in tumor bearing mice. *Br. J. Cancer*, **53**, 615.

KUMMERMEHR, J. & TROTT, K.R. (1982). Rate of repopulation in a slow and fast growing mouse tumour. In *Progress in Radio-Oncology*, Kärcher *et al.* (eds), Raven: New York, 299.

MERRY, S., FETHERSTON, C.A., KAYE, S.B., FRESHNEY, R.J. & PLUMB, J.A. (1986). Resistance of human glioma to adriamycin *in vitro*: the role of membrane transport and its circumvention by verapamil. *Br. J. Cancer*, **53**, 129.

MERRY, S., FLANIGAN, P., SCHLICK, E., FRESHNEY, R.J. & KAYE, S.B. (1989). Inherent adriamycin resistance in a murine tumour line: circumvention with verapamil and norverapamil. *Br. J. Cancer*, **59**, 895.

MEW, D., WAT, C.K., TOWERS, G.H.N. & LEVY, J.G. (1983). Photoimmunotherapy: treatment of animal tumours with tumour specific monoclonal antibody-hematoporphyrin conjugates. *J. Immunol.*, **3**, 1473.

NELSON, J.S., LIAW, L.H., ORENSTEIN, A., ROBERTS, G.W. & BERNS, M.W. (1986). Mechanism of tumor destruction following photodynamic therapy with hematoporphyrin derivative, chlorin and phthalocyanine. *JNCI*, **20**, 1599.

POTTER, W.S., MANG, T.J. & DOUGHERTY, T.J. (1987). The theory of photodynamic dosimetry: consequences of photodestruction of sensitizers. *Photochem. Photobiol.*, **46**, 97.

SAS-INSTITUTE, INC. (1985). *SAS Users Guide: Basics and Statistics.* Vers.5 ed., chapt 54, Cary, North Carolina.

SCHMIDT, W.F., HUBER, K.R., ETTINGER, R.S. & NEUBERG, R.W. (1988). Antiproliferative effect of verapamil alone on brain tumor cells *in vitro*. *Cancer Res.*, **48**, 3617.

SELMAN, S.H., KREIMER-BIRNBAUM, M., KLAUNIG, J.E., GOLDBLATT, P.J., KECK, R.W. & BRITTON, S.L. (1984). Blood flow in transplantable bladder tumors treated with hematoporphyrin derivative and light. *Cancer Res.*, **44**, 1924.

SROKA, R., GIEDL, J., GOSSNER, L. & 5 others (1989*a*). Photodynamic therapy of human gastrointestinal carcinomas: An *in vivo* study on the relationship between applied energy density and tumor destruction in a nude mouse model. *Laser Med. Surg.*, **2**, 111.

SROKA, R., ELL, C., GOTTSCHALK, W., HENGST, J. & UNSÖLD, E. (1989*b*). Homogenous light application and monitoring of the applied power density during PDT. *J. Photochem. Photobiol.*, **3**, 456.

STAR, W.M., MARIJNISSEN, H.P.A., VAN DEN BERG-BLOK, A.E., VERSTEG, J.A.C., FRANKEN, K.A.P. & REINHOLD, A.S. (1986). Destruction of rat mammary tumor and normal tissue microvascularisation by hematoporphyrin derivative photoradiation observed *in vivo* sandwich observation chambers. *Cancer Res.*, **46**, 2532.

STOCKER, S. (1986). *Ein Tumormodell der Maus zur Quantifizierung der photodynamischen Therapie*. Diplomarbeit, Universität München.

THOMAS, J.P. & GIROTTI, A.W. (1989). Glucose administration augments in vivo uptake and phototoxicity of the tumor-localizing fraction of hematoporphyrin derivative. *Photochem. Photobiol.*, **49**, 241.

TSURUO, T., LIDA, H., TSUKAGOSHI, S. & SAKURAI, Y. (1983). Potentiation of vincristine and adriamycin effects in human hemopoetic tumor cell lines by calcium antagonists and calmodulin inhibitors. *Cancer Res.*, **43**, 2267.

WEISHAUPT, K.R., GOMER, C.J. & DOUGHERTY, T.J. (1986). Identification of singlet oxygen as the toxic agent in photoinactivation of a murine tumor. *Cancer Res.*, **36**, 2326.

WIEMAN, T.J., MANG, T.S., FINGAR, V.H. & 6 others (1988). Effect of photodynamic therapy on blood flow in normal and tumor vessels. *Surgery*, **104**, 512.

WOOTON, R.S., SMITH, K.G., AHLQUIST, D.A., MULLER, S.A. & BALM, R.K. (1988). Prospective study of cutaneous phototoxicity after systemic hematoporphyrin derivative. *Las. Surg. Med.*, **8**, 294.


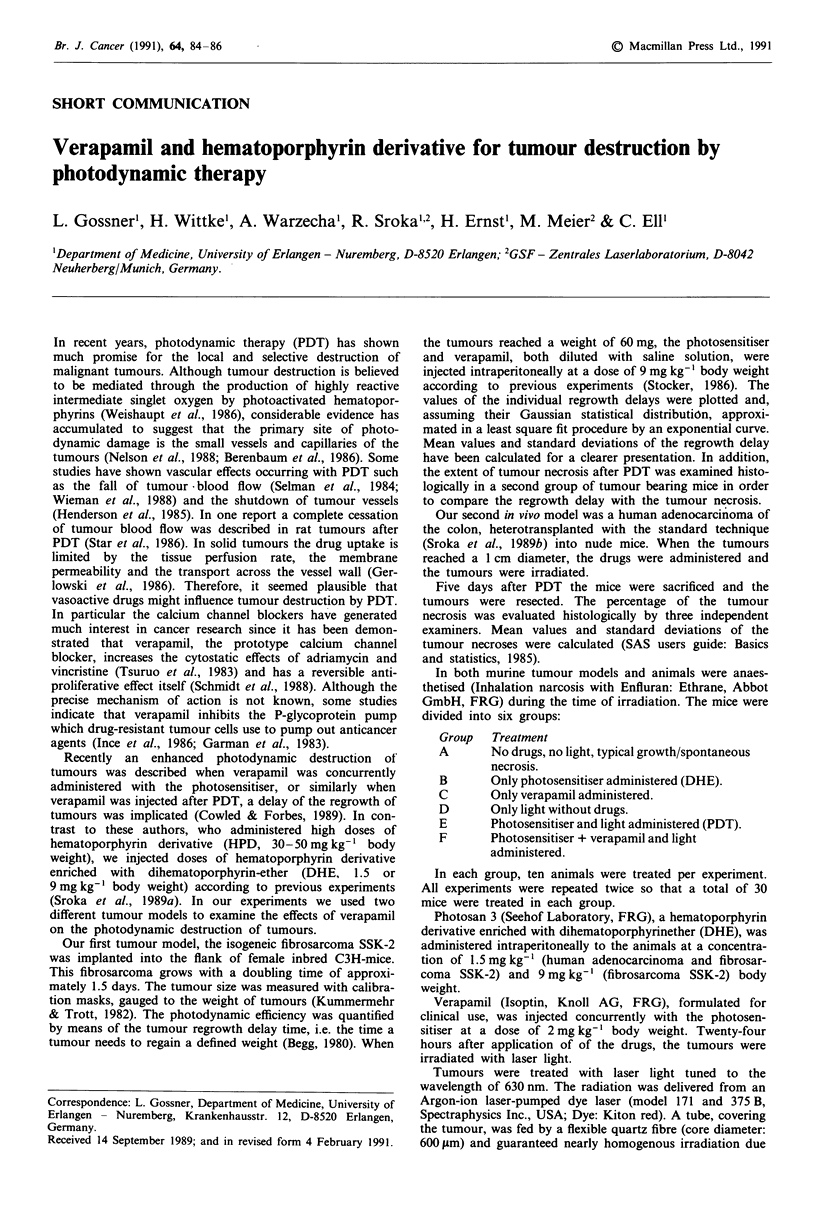

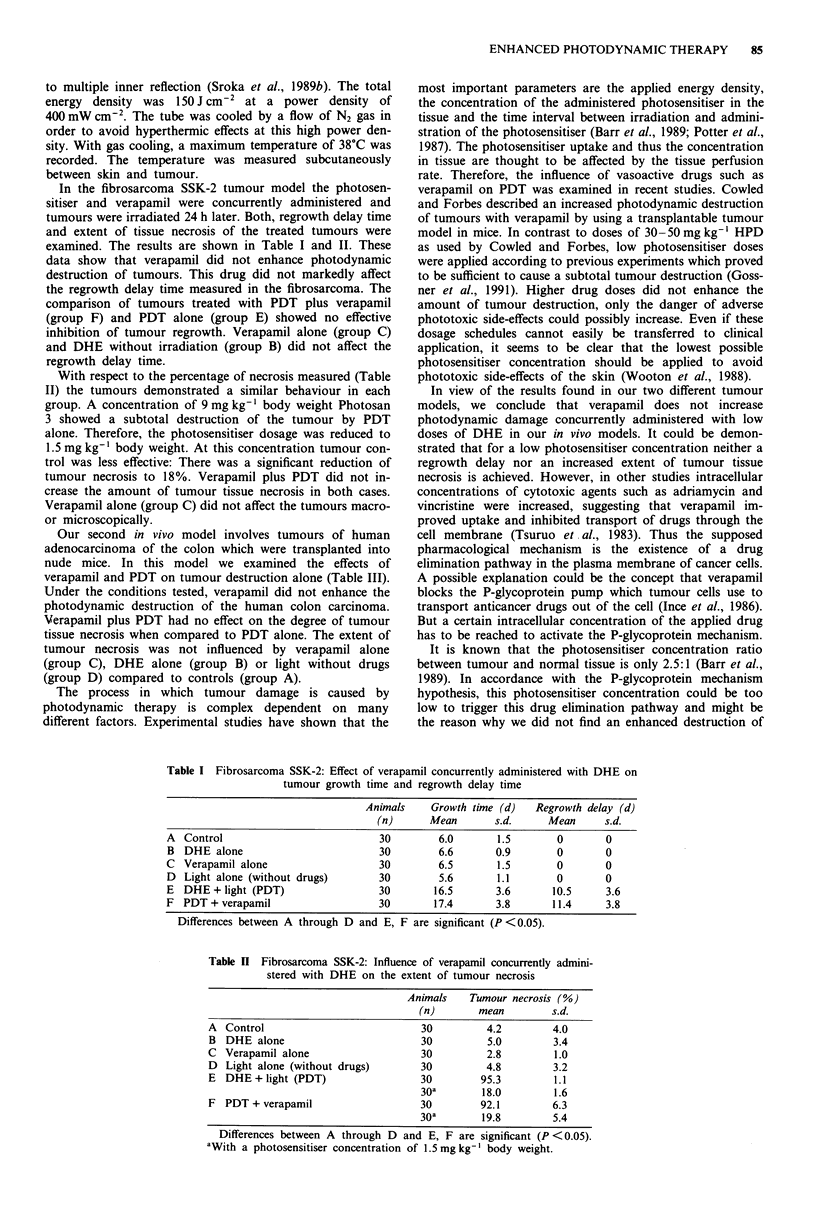

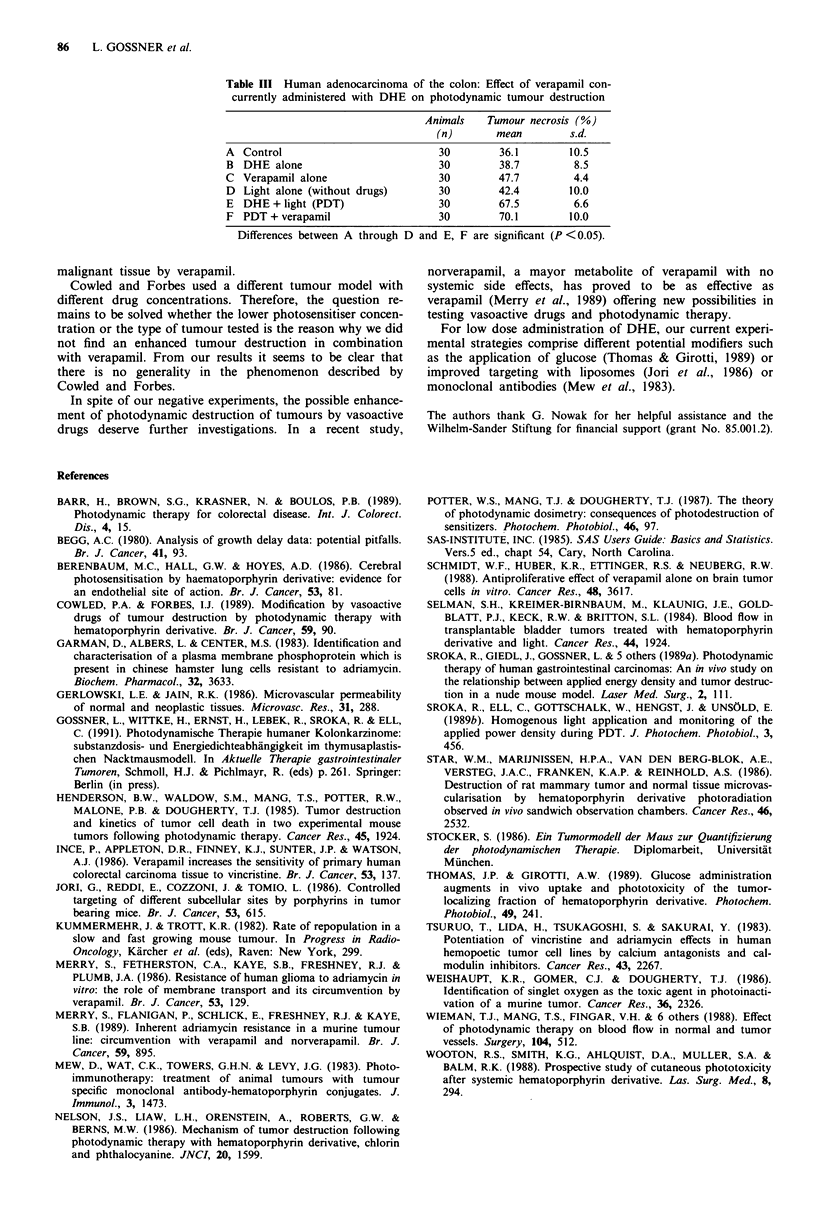


## References

[OCR_00336] Barr H., Bown S. G., Krasner N., Boulos P. B. (1989). Photodynamic therapy for colorectal disease.. Int J Colorectal Dis.

[OCR_00341] Begg A. C. (1980). Analysis of growth delay data: potential pitfalls.. Br J Cancer Suppl.

[OCR_00345] Berenbaum M. C., Hall G. W., Hoyes A. D. (1986). Cerebral photosensitisation by haematoporphyrin derivative. Evidence for an endothelial site of action.. Br J Cancer.

[OCR_00355] Garman D., Albers L., Center M. S. (1983). Identification and characterization of a plasma membrane phosphoprotein which is present in Chinese hamster lung cells resistant to adriamycin.. Biochem Pharmacol.

[OCR_00361] Gerlowski L. E., Jain R. K. (1986). Microvascular permeability of normal and neoplastic tissues.. Microvasc Res.

[OCR_00378] Ince P., Appleton D. R., Finney K. J., Sunter J. P., Watson A. J. (1986). Verapamil increases the sensitivity of primary human colorectal carcinoma tissue to vincristine.. Br J Cancer.

[OCR_00382] Jori G., Reddi E., Cozzani I., Tomio L. (1986). Controlled targeting of different subcellular sites by porphyrins in tumour-bearing mice.. Br J Cancer.

[OCR_00392] Merry S., Fetherston C. A., Kaye S. B., Freshney R. I., Plumb J. A. (1986). Resistance of human glioma to adriamycin in vitro: the role of membrane transport and its circumvention with verapamil.. Br J Cancer.

[OCR_00398] Merry S., Flanigan P., Schlick E., Freshney R. I., Kaye S. B. (1989). Inherent adriamycin resistance in a murine tumour line: circumvention with verapamil and norverapamil.. Br J Cancer.

[OCR_00404] Mew D., Wat C. K., Towers G. H., Levy J. G. (1983). Photoimmunotherapy: treatment of animal tumors with tumor-specific monoclonal antibody-hematoporphyrin conjugates.. J Immunol.

[OCR_00416] Potter W. R., Mang T. S., Dougherty T. J. (1987). The theory of photodynamic therapy dosimetry: consequences of photo-destruction of sensitizer.. Photochem Photobiol.

[OCR_00425] Schmidt W. F., Huber K. R., Ettinger R. S., Neuberg R. W. (1988). Antiproliferative effect of verapamil alone on brain tumor cells in vitro.. Cancer Res.

[OCR_00432] Selman S. H., Kreimer-Birnbaum M., Klaunig J. E., Goldblatt P. J., Keck R. W., Britton S. L. (1984). Blood flow in transplantable bladder tumors treated with hematoporphyrin derivative and light.. Cancer Res.

[OCR_00442] Sroka R., Ell C., Gottschalk W., Hengst J., Unsöld E. (1989). Homogeneous light application and monitoring of the applied power density during PDT.. J Photochem Photobiol B.

[OCR_00448] Star W. M., Marijnissen H. P., van den Berg-Blok A. E., Versteeg J. A., Franken K. A., Reinhold H. S. (1986). Destruction of rat mammary tumor and normal tissue microcirculation by hematoporphyrin derivative photoradiation observed in vivo in sandwich observation chambers.. Cancer Res.

[OCR_00461] Thomas J. P., Girotti A. W. (1989). Glucose administration augments in vivo uptake and phototoxicity of the tumor-localizing fraction of hematoporphyrin derivative.. Photochem Photobiol.

[OCR_00467] Tsuruo T., Iida H., Tsukagoshi S., Sakurai Y. (1983). Potentiation of vincristine and Adriamycin effects in human hemopoietic tumor cell lines by calcium antagonists and calmodulin inhibitors.. Cancer Res.

[OCR_00473] Weishaupt K. R., Gomer C. J., Dougherty T. J. (1976). Identification of singlet oxygen as the cytotoxic agent in photoinactivation of a murine tumor.. Cancer Res.

[OCR_00478] Wieman T. J., Mang T. S., Fingar V. H., Hill T. G., Reed M. W., Corey T. S., Nguyen V. Q., Render E. R. (1988). Effect of photodynamic therapy on blood flow in normal and tumor vessels.. Surgery.

[OCR_00483] Wooten R. S., Smith K. C., Ahlquist D. A., Muller S. A., Balm R. K. (1988). Prospective study of cutaneous phototoxicity after systemic hematoporphyrin derivative.. Lasers Surg Med.

